# Effect of user preferences on ITN use: a review of literature and data

**DOI:** 10.1186/s12936-017-1879-8

**Published:** 2017-06-01

**Authors:** Hannah Koenker, Joshua O. Yukich

**Affiliations:** 1 Johns Hopkins Center for Communication Programs, Baltimore, MD USA; 2 Center for Applied Malaria Research, Tulane University School of Public Health, New Orleans, LA USA

**Keywords:** Malaria, Bed net, Long-lasting insecticidal net, Insecticide-treated net, Preferences, Net use

## Abstract

**Background:**

Insecticide-treated bed nets (ITNs) are the primary tool for vector control, and optimizing ITN use is a key concern of national programmes. Available evidence indicates that bed net users often have preferences for shape, colour, size, and other attributes, but it is unclear whether these preferences are strong enough to have any significant effect on bed net use, and whether countries and donors should invest in more expensive attributes in order to maximize ITN use. The link between bed net attributes, preferences, and use was investigated using a literature review and review of publicly available, nationally representative household surveys from sub-Saharan Africa.

**Methods:**

A literature search was conducted to identify publications with data on preferences for net attributes and on associations between net attributes and use. Publicly available DHS and MIS datasets were screened for variables on net preferences and net attributes. Wald tests were run to obtain odds ratios and confidence intervals for the use of nets of various attributes in univariate analysis. A multilevel logistic regression was constructed to assess the odds of a net’s use, controlling for background variables and adding random effects variables at the household and cluster level.

**Results:**

Preferences for certain net attributes exist, but do not impede high rates of net use in countries where data were available. Stated preferences for shape and colour do not significantly influence net use to degrees that would require action by programme planners. By and large, people are using the nets they receive, and when they do not, it is for reasons unrelated to shape and size (primarily perceived mosquito density, heat or an excess of nets). Households in higher wealth quintiles tend to own greater numbers of conical nets, indicating that they have the ability to obtain or purchase these nets on their own, and individuals resident in higher wealth quintile households also use conical nets preferentially.

**Conclusions:**

The increased manufacturing costs for conical nets are not outweighed by the very small, often non-existent, increases in use rates in sub-Saharan Africa. Programmes that wish to explore the relationship between net attributes, preferences and use rates should include these questions in nationally representative household surveys to be able to capture trends across geographic and socio-economic groups.

**Electronic supplementary material:**

The online version of this article (doi:10.1186/s12936-017-1879-8) contains supplementary material, which is available to authorized users.

## Background

While it is well known that consumer preferences influence spending habits and use of commercial products, very little is known about the influence of preferences for mosquito bed nets on their use. Many studies have investigated determinants of use and barriers to use, with strong evidence that insecticide-treated net (ITN) access is the primary driver of ITN use [1–4], and that the primary barriers to use when ITNs are available are discomfort (heat) and low perceived mosquito density [5].

During the early days of treated bed nets, formative research identified preferred net attributes in an effort to maximize use by the target populations. From about 1980–2005, nets were treated and retreated with treatment kits, making it possible for consumers to purchase their preferred untreated net, and turn it into a treated net. With the introduction of mass campaigns and routine distribution, first in 2004/2005 to children under five and pregnant women, and then universal coverage distributions beginning in 2009, large-scale procurements of long-lasting insecticidal nets (LLINs), where treatment was done at the factory level, significantly reduced local net markets in sub-Saharan Africa [6]. Beneficiaries then began to receive nets of a particular colour, shape and size during mass campaigns and were not able to select a campaign net that might meet their preferences.

The logistics of large-scale procurement and distribution make it impossible to satisfy the variety of net preferences (for shape and colour, for example) that exist among consumers, as this would involve stocking enough nets of various types at all distribution points for people to be able to choose among them, requiring overly excessive procurement, or direct to beneficiary distribution system. On the other hand, programmes want to procure nets that will be most used and avoid procuring nets that may go unused for sleeping, due to procurement specification decisions. Programmes and donors also want to know whether differences in size, colour and shape, particularly, are worth additional investment in order to maximize net use. Previous work on the issue of value for money in LLIN procurement is summarized in a 2012 report from the Results for Development Institute, which examined the costs of colour, size, packaging, labels, and hanging materials [7]. Its conclusions: recommending increased standardization in height and width, bulk packaging, simplified packaging logos, standard labels, and not procuring additional hanging materials, have contributed to the development of standard LLIN specification in tenders amongst the major procurers. It did not, however, address shape, given the minimal data available, although it did note that in Mozambique, users who did not have their preferred net were no less likely to have used it than those who did have their preferred net. A 2013 study on bed net preferences in the Peruvian Amazon [8] called for net preference to be brought into the value for money equation, and for appropriate methods for studying and measuring net preference to be developed.

Current procurement practices of major donors (the Global Fund, US President’s Malaria Initiative (PMI), UNICEF) allow for some level of preference to be included in tenders, in varying ways. At the Global Fund, interim guidance has been developed [9] for all LLIN procurement (pooled or direct procurement), noting that all LLINs procured with Global Fund resources should have an interim or full WHOPES recommendation, have a maximum height of 180 cm, and be rectangular. They note that both polyester and polyethylene should be included to allow fair and open competition. For PMI, their 2015 technical guidance [10] refers to a recently defined ‘standard specifications’, and requests countries to provide justification for procuring non-standard LLINs. Manufacturers are asked in these cases to provide quotes for both the standard and non-standard options. UNICEF procures on behalf of governments and donors, meaning that once the specifications are agreed upon with PMI or Global Fund, UNICEF then issues tenders. They also have standard size options for rectangular nets in white, blue or green. If requests for conical or non-standard nets are received, these are discussed with the Ministry of Health, and may be accepted if the donor also agrees. In all cases, brand-specific requests are not allowed.

Since the advent of universal coverage campaigns, it has been difficult to even measure the influence of net attributes on net use, as the majority of nets in a given country are usually the same product. Few Demographic and Health Surveys (DHS) or Malaria Indicator Surveys (MIS) include even basic information on the net shape, size and colour that allow such analysis; even fewer include questions on the respondent’s preferences for various attributes.

This paper reviews the published literature on net use and user preferences, and examines the available data to assess whether shape, colour and size are influencing rates of net use. The extent to which these differences would affect programmatic decision-making is discussed.

## Methods

For the literature search, a PubMed search was done on the following terms: net, prefer*, malaria; net, malaria colour/colour; net malaria size; net malaria shape. Resulting publications were then screened, and included if they contained data on user preferences for nets, or data on net attributes as determinants of net use. References lists were then scanned for additional publications. The resulting 33 references were then reviewed and categorized (Additional file 1) as ‘acceptability’ studies, ‘crossover acceptability’ studies, ‘simple preference’ (reporting solely on stated preferences without including net attribute information), and ‘determinants of use’ (where net attributes were included as an independent variable, but preferences were not recorded). Full text records were obtained.

For the secondary data analysis, all publicly available DHS and MIS datasets since 2005 (n = 67) were obtained from the DHS website [11] and screened for variables related to the shape and colour of nets and any net preference variables (Table 1). Twelve datasets were found with data on net shape, from seven countries (Burkina Faso, Gambia, Malawi, Mali, Nigeria, Rwanda, Senegal). Four datasets contained information on respondents’ stated preferences for shape and/or colour, but did not contain information on shape or colour of nets in the survey households (Guinea, Kenya, Swaziland, Madagascar). Two datasets from Malawi contained both preference data and net shape and colour data.

**Table 1:** Net attributes and preference variables included in DHS and MIS

	Net shape	Net colour	Net size	Preferred shape	Preferred colour	Preferred size
Burkina Faso 2014	x					
Gambia 2013	x					
Malawi 2010	x	x		x	x	
Malawi 2012	x	x		x*	x*	
Malawi 2014	x	x		x	x	
Mali 2015	x					
Nigeria 2015	x					
Rwanda 2010	x					
Rwanda 2013	x				x	
Rwanda 2014	x					
Senegal 2008	x	x	Width			
Senegal 2010	x		Width			
Guinea 2012				x		
Kenya 2015				x	x	
Swaziland 2006**					x	
Madagascar 2013				x	x	Height

* In Malawi 2012, preferred shape and colour were only asked in households that did not own any ITNs

** In Swaziland 2006, preferred colour was asked only of households who reported they would like an additional ITN

Information on basic demographics and net characteristics for each net in the household was extracted and reshaped to form a ‘net file’ to permit analysis by net. Using information about the net brand, a ‘textile’ variable was created for selected datasets. A variable for household supply of nets was generated and coded as ‘not enough’ if the household owned fewer than one net for every two people; ‘universal coverage’ if the household owned at least one net for every two people, but fewer than one net per person; and ‘too many’ if the household owned at least one net for every household member. Single person households owning one net were classified as having ‘universal coverage’. Additional information was included in the 2010 and 2014 Malawi MIS. The household respondent’s preference for shape and colour was recorded, along with the reasons they preferred a particular shape in the 2014 MIS. A preference variable was created to assess whether the net being the preferred colour, shape, or both, was associated with increased use. The 2012 Malawi MIS recorded the preferred shape only among households that had no nets at all, making analysis of the relationship between the shape of the household’s nets, their use, and preferences impossible.

Using the ‘svy’ family of commands in Stata 14, the proportions of nets of various attributes were calculated, along with the per cent of nets that were used, disaggregated by attribute. Wald tests were run to obtain odds ratios and confidence intervals for the use of nets of various attributes in univariate analysis. Finally, building on work by Baume et al. [12, 13], other predictors of net use including basic demographic information (household supply of nets, age of head of household, sex of head of household, wealth quintile, urban/rural status, whether household was sprayed in the previous 12 months) and other net characteristics (age of net, net treatment status, presence of holes, shape, colour if available) were included in a multilevel logistic regression to assess whether shape remained a significant factor in the net’s use, controlling for these other variables. To correct for clustering, random effects at the household and cluster level were included in the model, and marginal effects were computed to obtain average marginal effects for net shape and for stated preferences where included in the datasets.

### Literature review

The 33 articles were divided into two categories: those that reported on stated preferences (preferences stated by study participants, whether through survey questionnaires or focus group discussions), and determinants of use (preferences demonstrated by increased use of specific types of nets). Twenty-six articles reported stated preferences, and seven reported determinants of use.

### Stated preferences

Early acceptability studies in The Gambia in the 1980s [14, 15] reported that the study population was already sleeping under traditional nets at very high rates (95% or greater), and assessed preferences for different textiles, with the majority preferring opaque sheeting to open netting, for privacy and durability. White was the preferred colour in these small studies (90%), washed two to three times a month to keep them clean [16]. With this information, a bed net trial using white nets made of sheeting fabrics was undertaken and reported on in 1993 [17], noting that 86% of subjects were bed net users. Use of nets during acceptability trials in northern Ghana was also high (99% in rainy and 20% in dry season), where 73% of respondents felt that the size of the nets was adequate, while the rest preferred a larger size [18, 19].

In the early 2000s, Tami et al. asked participants whether they preferred the Olyset or polyester nets when offered at the same price. Preferences were evenly split (51% preferred Olyset, 95% CI 41–61), and participants stated stronger preferences for green nets, to mask dirt [20]. An acceptability trial in India and Nepal also compared polyester and polyethylene nets, using a sequential design in India and a crossover design in Nepal (households used each net for 7 days total). There was a slight preference for the softer quality of polyester. Qualitative data noted varying colour preferences between men (green) and women (blue) [21].

In the late 2000s, a study in Sri Lanka explored preferences for shape among various ethnic/religious groups [22]. The majority preferred conical nets, although disaggregation by ethnic/religious group showed that Tamils liked the shapes they owned, 78% of Muslims liked their rectangular nets (none owned conical), and Sinhalese with conical nets strongly liked them, while only 37% of Sinhalese with rectangular nets liked them. The majority (82 and 76%) of Sinhalese and Muslims used their nets daily, while Tamil used them mainly in periods of high mosquito density.

Bed net acceptability trials in the Solomon Islands began with a qualitative study in 2009 to elicit preferences; respondents preferred darker (green) nets and wider nets to accommodate sleeping mats made of coconut leaves [23]. The ensuing crossover acceptability trial compared Olyset, PermaNet and DuraNet in a three-stage crossover design, with participants using each net for 10 days. Olyset were deemed less acceptable due to wrinkling and shrinking after washing; larger nets and darker colours were preferred, and shape preference was split equally between conical and rectangular [24]. A similar study in Vanuatu noted preferences for larger mesh and wider nets [25], while a qualitative study in Timor Leste, despite aiming to elucidate preferences, found that distinctions between attributes were muddied due to different brands having the same colour, and because participants did not have enough experience with nets to make preferences revealing [26].

In East Africa, a 2009 study explored preferences among 400 households in Central Kenya; 63% preferred rectangular nets, although this varied among the four villages in the sample. Green nets were the most preferred (51%) versus blue (21%), once again due to green’s dirt-concealing abilities. In the population, 96% of those with access to a net used one [27]. In Liberia, an acceptability trial of Interceptor nets noted that perceived insecticidal activity was an important factor for acceptability and use, and that despite half the respondents preferring blue, white ITNs were still used [28].

During the universal coverage campaign era, a study in Chipinge District, Zimbabwe specifically explored shape preferences. Respondents preferred conical ITNs (84%) compared to rectangular ITNs (15%), primarily due to easier hanging. The authors noted that conical nets were introduced 10 years prior to the rectangular nets, likely influencing overall preferences. In the study population of 380 matriarchs, 93% had received an ITN 2 years prior, while only 33% used one the previous night. No information on shape of nets in these households was gathered [29].

Two qualitative studies from the Peruvian Amazon found that LLINs were used less frequently in ‘open’ houses (with limited walls, no ceilings, open eaves), because they provided less privacy than traditional opaque nets, did not protect from the cold, and did not prevent debris falling onto beds from the roofing material [8, 30].

A 2014 study in Amhara, Ethiopia found that shape preference was more or less evenly split, with ease of use the main reason for preferring conical nets, and better fit with the bed the main reason for preferring rectangular nets. Blue colour and medium size were the most preferred [31]. However, in southwestern Ethiopia, conical nets were perceived to fit beds and houses better [32]. Qualitative work in Senegal found that while participants stated preferences for shape, size and colour, they did not feel that not having these attributes prevented use. Between 72 and 86% of nets in the study households were used the previous night [33]. An acceptability trial of Dumuria nets in Garissa, Kenya, confirming earlier qualitative findings from southern Sudan [34], found that non-mesh, opaque, bed-sheet-like fabric nets were accepted by 95% of participants from nomadic communities (81% if missing values are considered negative answers), and that darker colours were preferred to hide dirt [35].

A study from Cambodia reported that respondents preferred soft, colorful market nets with small mesh over coarse, small, LLINs with larger mesh [36]. These findings were echoed in a willingness-to-pay study in Laos [37] and in Madagascar [38].

From the published literature, the reasons behind preferences of various net attributes are summarized in Table 2.

**Table 2:** Reasons for preferring net attributes from qualitative data

Attribute	Perceived benefits
Rectangular	Fit more sleepers (Ng’ang’a [27], Kenya) Fits rectangular beds/sleeping areas (Sande [29], Zimbabwe; Beer [39], Zanzibar) Reduces body contact with the net (Sande [29], Zimbabwe) Easy to hang (Aleme [31], Ethiopia)
Conical	Easier to hang (Baume [12], Ethiopia; Banek [28], Liberia; Sande [29], Zimbabwe; Gobena [41], Ethiopia; Berthe [33], Senegal; Beer [39], Zanzibar; Banek [28], Liberia) More compatible with sleeping arrangements and house style (Birhanu [32], Ethiopia)
White	Looks clean (MacCormack [16], Gambia)
Green/blue	Dark colour masks dirt and smoke (Tami [20], Tanzania; Ng’ang’a [27], Kenya; Atkinson [24], Solomon Islands; Banek [28], Liberia; Gore-Langton [35], Kenya; Harvey [30], Peruvian Amazon; Gobena [41], Ethiopia; Gyapong [19], Ghana)
Larger size	Fits more sleepers (Gyapong [19], Ghana Binka [18], Ghana; Atkinson [25], Vanuatu; Shirayama [37], Laos) Less movement restriction (Atkinson [25], Vanuatu) Better ventilation (Atkinson [25], Vanuatu)
Polyethylene/larger mesh size	Good ventilation (Tami [20], Tanzania; Beer [39], Zanzibar) Stronger (Lover [26], Timor-Leste; Beer [39], Zanzibar)
Polyester/smaller mesh size	Softer (Tami [20], Tanzania; Mattern [38], Madagascar; Das [21], India) Warmer (Mattern [38], Madagascar) Keeps insects out better (Gryseels [36], Cambodia; Beer [39], Zanzibar; Atkinson [23], Solomon Islands; Das [21], India)
Opaque fabric	Provides privacy (MacCormack [14], Gambia; Peeters Grietens [8], Peruvian Amazon; Harvey [30], Peruvian Amazon; Bean [34], S Sudan) Keeps dust/debris/insects/spiritual forces out (MacCormack [14], Gambia; Peeters Grietens [8], Peruvian Amazon; Harvey [30], Peruvian Amazon) Attractive (MacCormack [14], Gambia) Warmer in cool weather (MacCormack [14], Gambia; Peeters Grietens [8], Peruvian Amazon; Harvey [30], Peruvian Amazon) Better at concealment from animals (Peeters Grietens [8], Peruvian Amazon; Bean [34], S Sudan)
Open netting fabric	Attractive (MacCormack [14], Gambia) Lighter weight and easier to wash (MacCormack [15], Gambia; Harvey [30], Peruvian Amazon) Cooler in hot weather (MacCormack [14], Gambia)

### Determinants of net use

In 2009, Baume et al. reported on a cross-sectional survey among 857 households in Oromia and Amhara States in Ethiopia. Net use with the net as unit of analysis was the outcome indicator. Conical ITNs were more likely to be used the previous night compared to rectangular ITNs [OR = 2.27 (95% CI 1.10–4.68) p < 0.05]. Colour of ITN was significant in the multivariate analysis, but it was removed from the final model because of collinearity with price (free/not free) and with shape (all ITNs distributed freely in the prior 2 years were blue rectangular). The researchers also noted that conical nets were easier to hang in the regions’ round houses. There were 1177 rectangular nets in the sample, with only 126 conical nets. Overall, 65% of nets were used the previous night [12]. A similar study in Ghana among 1796 households found that shape was not significant, while light blue colour (n = 332) was predictive of use compared to white colour (n = 1176), controlling for other net characteristics. Light blue nets were distributed for free in some areas and available in markets in others [13].

In Zanzibar, cross-sectional surveys were implemented in two districts among 509 caretakers of young children. Liking the net’s colour, size and shape of the LLIN were not significant factors in use of the net the previous night by children under-five. Over 85% of nets were used the previous night by a child under five. Qualitative data collected found that the community liked the light blue colour, but disliked the large mesh size and short height of the nets. Caretakers liking the mesh size was associated with increased use of the nets in one district, but not the other [39].

Two studies did not contain enough data to draw conclusions. A study in two villages (142 households) outside Kinshasa attempted to analyse net use by colour, but since only one net in the sample was not white, no conclusions could be drawn. ITN use by children age five to 15 was 90%, and just under 50% for adults. Shape was not recorded [40]. A study from eastern Ethiopia used a cross-sectional design among 2867 households and multivariate regression to determine reasons for non-use of LLINs [41]. ‘ITN colour preference’ was found to be a significant predictor, but was undefined in the publication. The authors stated that blue and cylindrical-shaped LLINs were preferable to white and rectangular, but showed no data for this.

In Zambia, a small cross-sectional survey using an MIS-style protocol among 483 households investigated the determinants of hanging and use of nets after a mass distribution [42]. Net characteristics, including shape, colour and whether or not the ITN was purchased, were not associated with net deployment, controlling for background variables.

A community-based, cross-sectional survey among 530 LLIN-owning households in Sri Lanka found that if the shape was conical, the odds of LLIN use were 5.6 times higher than if the shape was rectangular. The nets were evenly split between Yorkool and Olyset nets, with 505 conical and 155 rectangular. The main reason for not using a net was due to heat or lack of mosquitoes; 79% of the nets were used the previous night. Other variables associated with use were more residents, fewer untreated nets, participants reporting practical benefits of LLINs over untreated nets (e.g., greater durability or ventilation), newer nets and lack of side effects [43].

### Summary of literature review

The nineteen studies reporting on stated preferences do not link actual net use to the stated preferences, either by design (e.g., an acceptability trial providing only one type of net, or directing net use for a certain time period) or by oversight. Stated preferences can be useful in gauging likes and dislikes, but only determinants of use tell how net use is affected (or not) by these preferences.

Among the seven studies that reported on determinants of use, five included adequate information to compare shape and/or colour as dependent variables in multivariate analysis. Of these five, two found a significant association between shape and net use, and three found no effect. Regarding colour, only one study found a significant effect of colour on net use, and three found no effect. Neither overall size nor height of net were studied.

## Secondary data analysis

### Stated preferences

#### Shape

Six datasets included information on shape preferences, among four countries, shown in Table 3. In Guinea, preferences were more or less evenly split among rectangular, conical, and ‘either’. Preferences in Madagascar were primarily for rectangular nets (70%). In Malawi, preferences were for conical nets, going from 62% in 2010, falling to 55% in 2012, and rising again to 73% in 2014. Preferences were split evenly in Kenya.

**Table 3:** Household stated preferences for shape

Survey	All households			Households owning no nets		
	% of households who prefer rectangular nets	% of households who prefer conical nets	% no preference	% of households who prefer rectangular nets	% of households who prefer conical nets	% no preference
Guinea DHS 2012	34.8	38.7	25.0			
Kenya MIS 2015	43.7	47.3	9.0			
Madagascar MIS 2013*	40. 9	15.9	43.3**			
Malawi DHS 2010	35.7	61.5		42.6	50.8	6.6
Malawi MIS 2012				43.7	55.1	1.2
Malawi MIS 2014	26.7	72.8	n/a	37.9	62.1	n/a

* Individual-level data

** An initial question asked if respondent had any preferences about nets; if yes, respondents were then asked questions about preferred shape (reported here), texture, colour, height, and brand. These questions were combined to produce these results

In Malawi 2014, participants were asked why they preferred specific shapes. These match closely with findings from other literature noted in Table 2: 49% reported that conical nets were easier to hang, compared to rectangular (23%); 29% reported that rectangular nets allowed more people to sleep under them, compared to conical (6%). Similar percentages (15%) of respondents reported that conical and rectangular nets fit beds better, look nicer (13–16%), and are stronger (9–11%).

When shape preferences were analysed sub-nationally, for stated preferences, three of eight regions in Guinea preferred rectangular nets, while four regions preferred conical, and one (Mamou) preferred conical and ‘don’t care’ equally. In Malawi in 2010, all three regions preferred conical nets roughly 60:40, and this continued in 2014, increasing to a roughly 70:30 ratio. In Kenya (2015), the Coast and Northeastern regions had strong preferences (>2:1) for conical nets, while the remaining provinces were evenly split between the two shapes, and households in Nairobi preferred rectangular nets.

**Fig. 1:**
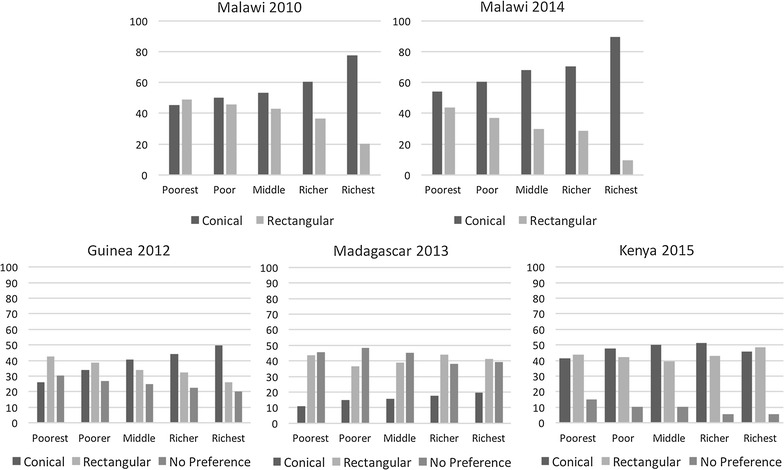
Net shape preferences by wealth quintile in five surveys

Preferences for conical nets were strongly and positively correlated with wealth quintile, with the exception of Kenya 2015 (Fig. 1; Additional file 2). In 2010 and 2014 Malawi surveys, preferences for shape were fairly evenly split at the lowest wealth quintile. In Guinea, the two lowest wealth quintiles preferred rectangular nets, while the upper three quintiles preferred conical to increasing degrees, and a significant percentage of households (20–30%) reported no preference. In Kenya, the middle three quintiles had a slight preference for conical nets, while the lowest and highest quintiles had slight preferences for rectangular nets. The proportion of those answering ‘no preference’ decreased with increasing wealth.

Female survey respondents were slightly more likely to prefer conical nets than male respondents. Shape preferences were not correlated with housing type. These findings are described further in Additional file 2.

#### Colour

**Fig. 2:**
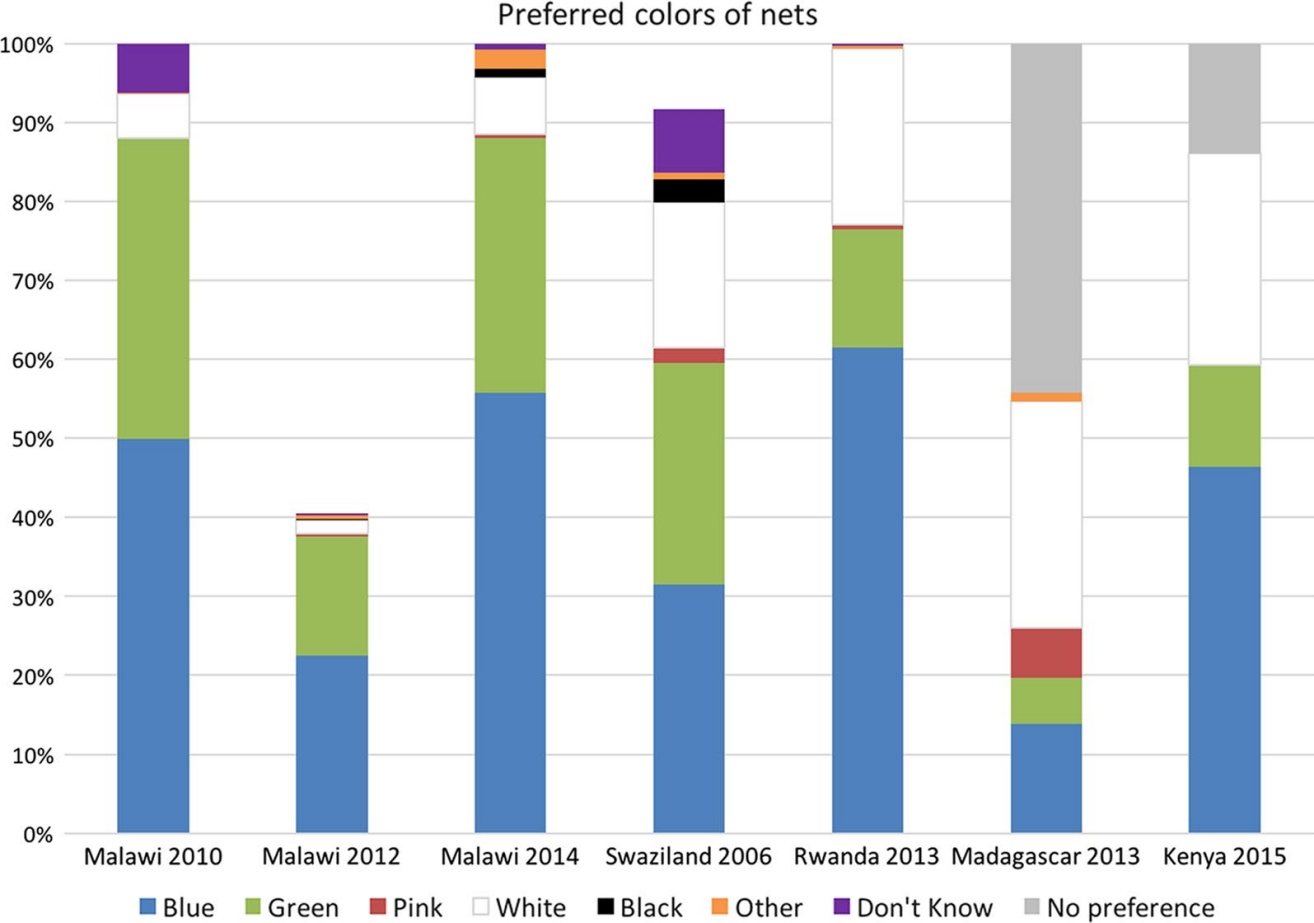
Stated preferences for net colour

Seven datasets contained information on respondents’ preferred colours for nets Fig. 2. Blue was the most popular colour in Malawi, Kenya, and Rwanda, followed by green. White was the preferred colour in Madagascar (2013), but was not popular in Malawi or Rwanda, and moderately popular in Swaziland (2006) and Kenya (2014). Missing values are included (Malawi 2012, Swaziland 2006) where significant; in Malawi 2012, the question was only asked in households owning zero nets; in Swaziland, the question was asked only in households that had indicated they needed additional nets.

#### Size and textile preferences

In Madagascar 2013, of the 5154 respondents who said they did have preferences about nets, 92% preferred taller nets (180 cm height) *versus* shorter nets (150 cm height); 85% preferred softer textile (polyester) over ‘hard texture’ polyethylene nets.

### Reasons for non-use or non-hanging of nets related to net attributes

Several surveys included additional questions to investigate the reasons for non-use of nets and/or for non-hanging of nets. Of particular interest are those that include net attributes as potential answer options. While the majority of nets were used the previous night in all countries (Table 4), the primary reasons for a net not being used were because they were extra and/or had not been used at all (Kenya 2015, 28%); the usual user was not at home that night (Kenya 2015, 21%); no mosquitoes (Senegal 2010, 68%; Senegal 2012, 68%; Senegal 2014, 63%). In the few cases where size, shape or colour were included as answer options, these comprised less than 2% of reasons why nets were unused or not hung in Kenya, and 0.01% in Burkina Faso.

**Table 4:** Percent of nets used the previous night, by shape

Survey	N (nets)	% of nets in the sample that are		% of nets that were used the previous night				Crude OR of rectangular net being used (vs conical)	p
		Conical	Rectangular	Total	Conical	Rectangular	Difference (% points)		
Burkina Faso MIS 2014	15,850	0.53	99.5	96.5	96.3	96.0	0.3	0.9	0.923
Gambia DHS 2013	13,528	64.9	35.1	74.5	75.8	72.0	3.8	0.8	0.019
Malawi DHS 2010	30,170	21.9	78.1	63.4	70.0	62.0	8.0	0.7***	<0.001
Malawi MIS 2012	3316	17.2	82.8	88.6	90.1	88.2	1.9	0.8	0.333
Malawi MIS 2014	4754	11.1	88.9	83.5	85.7	83.2	2.5	0.8	0.402
Mali MIS 2015	15,198	2.4	97.6	98.3	97.6	98.4	−0.8	1.5	0.313
Nigeria MIS 2015	12,637	3.8	96.2	60.5	64.0	60.3	3.7	0.9	0.398
Rwanda DHS 2010	20,825	51.0	49.0	68.7	67.4	70.2	−2.8	1.1***	<0.001
Rwanda MIS 2013	7962	48.2	51.5	72.4	79.6	65.6	14.0	0.5***	<0.001
Rwanda DHS 2014	20,448	81.6	18.4	77.6	78.4	74.0	4.4	0.8***	<0.001
Senegal MIS 2008	22,058	14.0	86.1	64.9	67.3	64.5	2.8	1.1	0.162
Senegal DHS 2010	22,285	34.0	66.0	69.5	68.8	69.8	−1.0	1.0	0.571

*** p < 0.001

### Modification of nets

Three surveys contained data on whether a net had been modified from its original shape. In Senegal 2012, 4.6% of nets were modified, increasing to 5.8% in the 2014 survey. The nature of the modification was not recorded. In Malawi 2014, 5.9% of the 588 conical nets had been altered from their original rectangular form to be conical. For three of these 35 nets, two nets were used to do the alteration; the remainder were altered using only the original net.

### Determinants of net use

#### Shape

Twelve datasets from seven countries include information on net shape (Table 4). In Malawi and Senegal, the majority of nets are rectangular in all surveys. Rwanda is more or less evenly split between the two shapes; The Gambia has primarily conical nets (2:1). Burkina Faso, Mali, and Nigeria had nearly all rectangular nets (96–99%).

If the sample is restricted to only households that own both types of nets (Table 5), a similar pattern is seen, with statistically significant differences for the same three countries (Malawi 2010, Rwanda 2010, 2013, and 2014), and greatest discrepancy in use rates in Rwanda 2013 of 24 percentage points.

**Table 5:** Per cent of nets used the previous night by shape, among households owning both shapes

	Conical	Rectangular	Difference (% points)	p	obs
Burkina Faso 2014	98	88	9	n/a	89
Gambia 2013	72	72	0	0.874	5797
Malawi 2010	73	66	7***	<0.001	6138
Malawi 2012	89	86	3	0.418	552
Malawi 2014	83	75	8	0.188	714
Mali 2015	98	98	0	0.992	1111
Nigeria 2015	71	74	−3	0.496	291
Rwanda 2010	60	64	−5**	0.005	5841
Rwanda 2013	78	54	24***	<0.001	2649
Rwanda 2014	73	67	6***	<0.001	3810
Senegal 2008	68	69	−1	0.639	5051
Senegal 2010	73	73	0	0.878	6530

** p < 0.01

*** p < 0.001

**Fig. 3:**
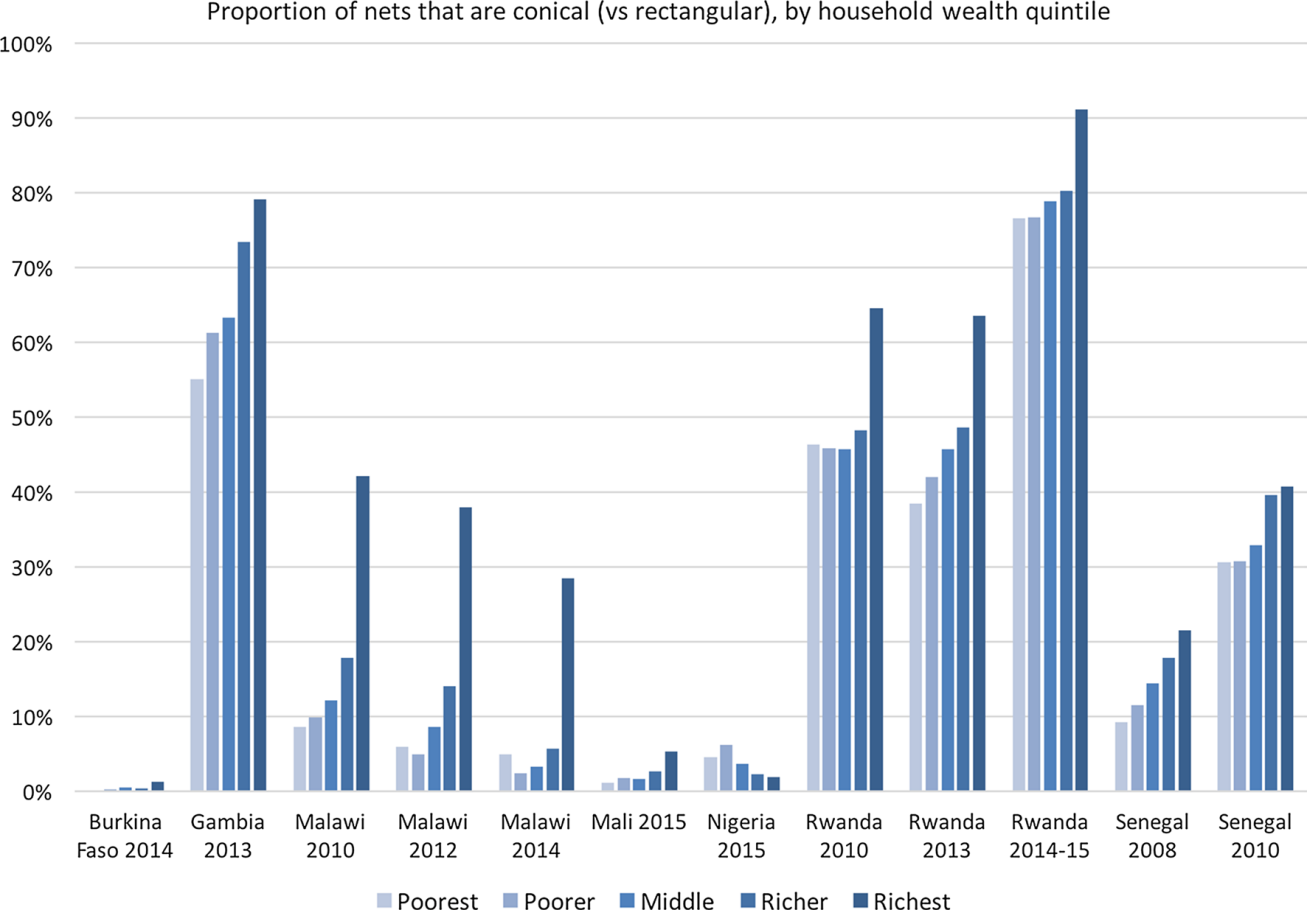
Proportion of conical nets owned by households in different wealth quintiles

In most countries, the proportion of nets that are conical (vs rectangular) increases with wealth quintile (Fig. 3), with pronounced differences in the highest wealth quintiles in Malawi and Rwanda.

To confirm that ownership of particular shapes of nets was not restricted to certain regions or wealth quintiles, the nine datasets were assessed. The overall percentage of net-owning households that owned both conical and rectangular nets ranged from 12.4% (Malawi 2012) to 37.9% (Gambia 2013), with some households owning both shapes in each area of the country (Additional file 3). Owning both shapes was positively associated with increasing wealth quintile (Additional file 3), with the exception of The Gambia.

Except for the Rwanda 2013 MIS, the per cent of all conical and rectangular nets that were used the previous night are programmatically equivalent, with differences of between 1 and 7 percentage points. However, in The Gambia, Malawi and Rwanda DHS datasets containing 20,000–30,000 nets, these small percentage point differences result in statistically significant differences in net use.

An alternative indicator of net use is mean number of users per nets of different shapes in all households and among households that own both shapes. This indicator takes into account nets that go unused and provides the basis for overall population protected when costs are taken into consideration. In Table 6, F is defined as the factor by which use increases for conical nets, here shown as the ratio of mean users per conical net to mean users per rectangular net, for each survey.

**Table 6:** Mean users per net for conical and rectangular nets

	Mean users per net, all households					Mean users per net in households owning both shapes				
	Con.	Rect	F (con./rect.)	p=	N	Con.	Rect	F (con./rect.)	p=	N
Burkina Faso 2014	1.31	1.66	0.79	0.141	15,850	1.37	1.32	1.04	0.929	107
Gambia 2013	1.60	1.40	1.14	<0.001	13,528	1.44	1.37	1.05	0.043	5768
Malawi 2010	1.30	1.36	0.96	<0.001	30,170	1.37	1.14	1.20	<0.001	6120
Malawi 2012	1.71	2.07	0.83	<0.001	3316	1.75	1.60	1.09	0.305	552
Malawi 2014	1.46	1.80	0.81	<0.001	4754	1.47	1.22	1.20	0.016	714
Mali 2015	1.61	1.72	0.94	0.05	15,198	1.57	1.62	0.97	0.603	1227
Nigeria 2015	1.21	1.19	1.01	0.941	12,637	1.29	1.40	0.92	0.271	292
Rwanda 2010	1.43	1.69	0.85	<0.001	20,825	1.34	1.49	0.90	<0.001	5851
Rwanda 2013	1.63	1.54	1.06	0.002	7962	1.82	1.19	1.53	<0.001	2668
Rwanda 2014	1.61	1.74	0.92	<0.001	20,448	1.64	1.45	1.14	<0.001	3811
Senegal 2008	1.30	1.27	1.02	0.098	22,058	1.29	1.26	1.02	0.152	5014
Senegal 2010	1.43	1.45	0.99	0.297	22,285	1.53	1.44	1.06	0.001	6555

In Table 6 the mean number of users per net is higher for conical nets in The Gambia 2013, Nigeria 2015, Rwanda 2013 and Senegal 2008 when looking at all households. However, when the sample is restricted to households that own both shapes, the mean number of users per conical net is higher in all surveys, except Rwanda 2010, Mali 2015, and Nigeria 2015. Given the higher ownership of conical nets in wealthier quintiles (Fig. 3), this may indicate preferential use of conical nets by households that can afford to pay for their preferred net.

In Malawi, 2010 and 2014 data on preferences were recorded for each household. In 2010, the use rate for nets that did not match the user preference for shape and colour was the same as the use rate for nets that did match shape and colour preference, both at 64%. Nor did use rates differ for nets that matched (64%) or did not match (63%) shape preference only.

In 2014, the use rate for nets that did not match the user preference for shape and colour was 83%, versus 87% for nets that did match both shape and colour preference. If the net matched only the shape preference, use rate was 86%, compared to 82% for nets that did not match the shape preference. Neither of these differences was statistically significant at the p < 0.05 level.

#### Textile

To check whether the textile makes a difference in use rates, the ‘brand’ variable was used to create a new variable indicating the material of the net. This was done only for the countries that also had information on shape although this analysis could be expanded, in principle, to other countries. Table 7 demonstrates that although there are some statistically significant differences in use rates of nets of various textiles (for surveys with over 20,000 nets), there are no significant programmatic differences in these rates. Of note, in the Rwanda surveys, the variable brand was unfortunately coded as a single value for any of the LLIN brands (i.e., PermaNet, MamaNet, TuzaNet, Olyset, NetProtect all shared a single code), and textile was not recorded separately.

**Table 7:** Percentage of nets used the previous night for different net textile types

	N (nets)	Polyester	Polyethylene	Cotton/DK	Polypropylene	P
Burkina Faso 2014	14,135	95.1	96.9	94.3		<0.001
Gambia 2013	13,940	74.3	76.5	76.3		0.693
Malawi 2010	30,718	66.5	59.4	63.0	69.4	<0.001
Malawi 2012	3315	88.6	89.7	83.9	86.9	0.065
Malawi 2014	4750	80.9	83.8	82.5	91.4	0.497
Mali 2015	13,942	98.3	98.6	98.8		0.392
Nigeria 2015	12,637	64.3	51.2	58.2		<0.001
Senegal 2008	22,496	63.9	68.0	64.6		0.350
Senegal 2010	22,218	69.4	68.7	70.1		0.895

#### Size

Only Senegal 2008 and 2010 recorded the size of net, in four categories (one place, two places, three places, ‘baby’ size). Table 8 shows the mean users and rates of use for each size in these surveys.

**Table 8:** Net use and size of nets in Senegal 2008 and 2010

	Mean users per net		% of nets used		Obs.	
	2008	2010	2008 (%)	2010 (%)	2008	2010
One-place (ref)	0.99	1.18	66	78	188	310
Two-place	1.21	1.33	62	68*	15,213	9993
Three-place	1.48*	1.56*	73	71	6561	11,774
Four-place		1.34		56*		224

* p < 0.05

### Determinants of net use: multivariate analysis

Building on work done by Baume et al. and MacIntyre et al. in Ethiopia, Ghana, and Zambia [12, 13, 42], regression models were developed using background characteristics of households and net attributes available in each dataset, to assess the influence of shape on net use, controlling for the household and net level fixed effects (household net supply, urban/rural residence, age and sex of the household head, socio-economic quintile; net shape, age, ITN status, colour, and preferences where available), and including random effects at both net and household level to correct for clustering (Table 9). The adjusted odds ratios indicate that net shape was a significant predictor of net use in the Malawi 2010, Malawi 2012, and the three Rwanda datasets. Net colour was not a significant predictor of net use in any dataset where it was present. The factors with the largest odds ratios predicting net use were urban residence (in The Gambia and Senegal), household supply (having too few nets greatly increased the odds of a particular net being used across all surveys, except Senegal 2008), and wealth quintile; odds of nets being used decreased with increasing wealth in Burkina, The Gambia, Nigeria, and Senegal 2010, but increased with increasing wealth in Malawi 2010, Rwanda 2010 and Rwanda 2014. Odds of net use increased in six surveys if the net was an ITN (vs untreated).

**Table 9:** Adjusted odds ratios for outcome ‘net was used the previous night’ in 12 surveys across seven countries in sub-Saharan Africa

Survey (obs)	Burkina 2014 (14,086)	Gambia 2013 (13,486)	Malawi 2010 (30,035	Malawi 2012 (3314)	Malawi 2014 (4750)	Mali 2015 (13,942)	Nigeria 2015 (12,601)	Rwanda 2010 (20,786)	Rwanda 2013 (7923)	Rwanda 2014 (20,313)	Senegal 2008 (21,951)	Senegal 2010 (22,193)
Household-level fixed effects												
Household net supply (ref: owns 1 net for 2 people)												
Not enough (<1 net for 2 people)	1.16	1.71***	1.37***	2.37***	2.23***	2.63***	1.08	1.98***	2.11***	1.98***	0.92	1.51***
Too many (>1 net for 2 people)	0.35***	0.22***	0.40***	0.14***	0.25***	0.22***	0.29***	0.44***	0.32***	0.31***	0.56	0.23***
Household is urban (vs rural)	0.89	8.76***	1.57***	2.19**	1.92*	0.77	0.88	1.20*	1.13	1.37**	27.72***	5.41***
Age of household head (ref: <5)												
35–49	2.32***	1.07	0.99	0.94	1.12	1.46	0.99	1.04	1.05	0.88*	0.86	0.79
50+	1.89**	1.12	0.95	1.15	0.88	1.30	1.18	1.31***	1.24*	1.15*	0.89	0.93
Household head is female	0.71	0.78	0.88*	1.12	0.91	2.95*	0.59***	1.08	1.07	1.13*	0.97	0.82
Wealth quintiles (ref: poorest)												
Poorer	0.50*	0.63*	1.12	0.77	1.36	0.95	0.86	1.25***	1.06	1.01	1.18	1.09
Middle	0.42**	0.51**	1.42***	1.21	1.96**	0.99	0.41***	1.43***	1.04	1.19*	1.00	0.94
Richer	0.45**	0.38**	1.46***	1.04	1.46	0.87	0.18***	1.62***	1.23	1.31***	2.31***	0.57*
Richest	0.24***	0.33**	1.70***	0.97	1.37	0.81	0.09***	1.81***	1.41**	1.38***	1.62	0.41**
Net-level fixed effects												
Net is conical (vs rectangular)	1.33	1.04	1.53***	1.95*	1.35	1.55	1.42	0.91*	2.26***	1.44***	0.95	1.04
Net age (ref: <1 year)												
1–2 years	1.19	0.56	1.23***	1.91**	1.77***	1.23	0.97	1.34***	1.50***	1.08	1.27*	1.15
2–3 years	0.61	1.05	1.14	1.46	1.53	1.39	1.02	1.38*	1.26	1.01	1.00	0.84
3+ years	0.90		0.97	1.06	0.69*	1.09	0.93	1.21**	1.57***	0.93	1.12	0.71*
Is an ITN (vs untreated)	2.61*	1.21	1.78***	2.74***	3.38***	1.38	1.14	1.36*	1.53	0.64	1.31*	1.04
Green (vs other colour)			1.05	1.11	0.47**							
Net matches stated preferences (ref: does not match)												
Both shape and colour			1.00		1.78*							
Only shape			1.08		1.20							
Only colour			1.06		1.09							
Net was purchased				1.11							1.63***	1.69***
Average marginal effects (percentage points by which proportion of nets used the previous night increase if all nets are conical, or all nets match shape/colour preferences												
Net is conical	0.6	0.3	5.9***	4.5*	2.8	0.7	4.0	−1.7*	13.1***	5.3***	−0.4	0.3
Net matches both preferences (vs neither)			0.0		5.2*							
Matches only shape (vs neither)			1.0		1.8							
Matches only colour (vs neither)			0.8		0.9							

* p < 0.05; ** p < 0.01; *** p < 0.001

In the 2010 and 2014 Malawi MIS, information on a household’s preferred shape and colour of net were recorded. Nets were then categorized into four groups: neither the preferred shape nor colour; both the preferred shape and the preferred colour; just the preferred shape (but not colour); and, just the preferred colour. In 2010, net use was not significantly different whether the net type matched the user preferences or not. In 2014, a net that was both the preferred colour and shape had 1.78 greater odds of being used the previous night. Average marginal effects were computed for each country. For net shape the results were statistically significant only in the Malawi and Rwanda datasets. In those datasets, the overall percentage of nets used the previous night have significantly improved if all nets in the dataset were switched to conical shape, by 5.9 and 4.5 percentage points in Malawi 2010 and 2012, respectively, and by 13.1 and 5.3% points in Rwanda 2013 and 2014, respectively. Average marginal effects were also computed for the preferences variables in the Malawi datasets, but were generally not statistically significant. The results for the preference variables were statistically significant in the Malawi 2014 dataset, where the proportion of nets used the previous night would have increased by 5% points if all nets had been the user’s preferred shape and colour.

### Cost implications

Costs for ITNs came down significantly in 2014–2015 as oil prices fell and competition among suppliers increased. Different net attributes have different cost implications. Standard colours (white, blue, green) have minimal cost differences, although white polyester nets are slightly cheaper than coloured nets, given the dyes and dying process involved. For polyethylene nets there is generally no price premium for colour, as additives for colour are needed even for white nets. The Value for Money report noted a price premium of US$0.79 for nets over 170 cm in height, and recommended that only at least double size (>130 cm wide) nets be procured to avoid limiting the number of sleepers [7].

The Global Fund’s most recent tender for LLINs conducted in 2015 provided a list of indicative prices for standard and custom LLINs as shown in Table 10. The actual costs will vary depending on factors such as volume; nets with non-standard specifications as shown in the Table are generally not purchased by the Global Fund but were requested for information only. Red text indicates non-standard options. PMI’s data on procurement costs have not been publicly updated since 2012, when costs of conical ITNs were on average $2.42 more expensive (ex-works) to procure than rectangular, or 1.54 times more expensive [44].

**Table 10:** Standard long-lasting insecticidal net reference prices from the Global Fund and from UNICEF Supply Division in 2016

Net type and dimensions (cm: L × W × H)	Global Fund standard LLIN* reference price 2016 (US$)		UNICEF Supply Division June 2016 75–100/110–150-denier (US$)
Rectangular			
180 × 160 × 150	2.11	2.35*	1.83/1.89
180 × 160 × 170	2.30		
180 × 160 × 180	2.29		
180 × 160 × 200	2.66		
Non-standard rectangular			
180 × 190 × 150	2.32	2.57*	1.99/2.09
180 × 190 × 160	2.43		
190 × 180 × 170	2.53		
190 × 180 × 180	2.64		
195 × 160 × 200	2.81		
200 × 140 × 150	2.26		
210 × 190 × 180	2.99		
Hammock 140 × 240	11.94		
+Net 240 × 65 × 120			
Conical			
1000 × 65 × 250	4.18	4.21*	
1050 × 65 × 220	3.99		3.04/3.29
1250 × 65 × 250	4.46		

The Global Fund reference prices include accessories such as hooks/strings and bags, are for 100-denier material, and are based on a range of prices from ten suppliers

The UNICEF prices are not directly comparable, as the net prices are separate from the accessories. These are reference prices only

* Simple average of Global Fund costs in matching cells (excluding hammock nets)

Conical nets are more expensive to manufacture as they require more skilled labourers for both cutting and sewing the trapezoidal panels, fixing the ring. Adjusting production lines for conical nets also has cost implications and sometimes implications for meeting production timelines. Conical nets require more fabric and raw materials (an additional 2–3 sq m of fabric, compared to rectangular nets, and the supporting ring). Due to their increased packing size and weight per net, conical nets can also be more expensive to transport and warehouse, as they can be up to twice the volume of rectangular nets [45]. For calendar year 2015, two of the 104 orders listed in the Global Fund PQR Database were for conical ITNs, comprising 2% of total ITNs purchased.

### Cost and utility functions

For most countries, gap analyses provide the number of ITNs required and volumes and, often, specifications are written into concept notes (in the case of the Global Fund) or Malaria Operational Plans (in the case of PMI). While there are not always fixed budgets from the outset countries are nonetheless faced with decisions about which net types to purchase. This can result in trade-offs whereby a more preferable net, which may be more likely to be used, is more expensive and therefore, given a fixed budget, fewer of these nets would be bought overall (or, alternatively, other components of the programme would be reduced). It is difficult currently to evaluate these trade-offs. However, this decision can be viewed as a basic optimization problem. For simplicity, a fixed budget is assumed. Two functions are needed: a cost function and a utility function. Here utility is defined as the total number of people using a net, using mean users per net.

#### Cost function

$$B = C_{o} N_{o} + N_{n} C_{n}$$

where B is the total budget, N_o_ is the number of standard nets to be purchased, C_o_ is the cost per standard net including delivery, N_n_ is the number of alternative nets to be purchased, and C_n_ is the cost per alternative net including delivery.

#### Utility function

$$T = N_{o} U + N_{n} (UF)$$

where T is the total number of people expected to sleep under nets, N_o_ is the number of standard nets, U is the mean number of persons sleeping under the standard net, N_n_ is the number of alternative nets to be purchased, and F is the factor by which use increases with the alternative net.

If the cost function is substituted and rearranged, the utility function can be rewritten as:

$$T = \left( {\frac{{B - N_{n} C_{n} }}{Co}} \right)U + N_{n} \left( {UF} \right)$$

for the purpose of reducing the dimensionality of the problem and to bring all the budget and utilization considerations into one equation.

The partial derivative of the utility function can then be taken in terms of the number of alternative nets purchased to yield a formula, which describes the direction of the change in the total number of net users as the number of alternative nets purchased increases.

$$\frac{{{\Delta}T}}{{{\Delta}N_{n} }} = UF - \left( {\frac{{C_{n} }}{{C_{o} }}} \right)U$$

If this last equation is evaluated at a given set of inputs, programmers can determine whether to switch or not: if the answer is positive, it is worthwhile to buy alternative nets, as the number of overall net users increases as a result. If the answer is negative, countries should procure the standard nets to maximize net users. If the answer is zero, there is no difference either way.

The final two equations here have been used in an Excel tool (Additional file 4) to further illustrate how programmes may decide to make evidence-based decisions about net procurement at different net costs. The tool provides space to make the inputs of mean users per net, cost and budget, and provides both the ‘yes/no’ answer about whether to switch to alternative nets, as well as the total number of net users expected with each type of net, and the expected loss or gain in net users if alternative or standard nets are procured.

### Case study for shape: Rwanda

To illustrate the above equations, Rwanda provides a case study. From the data presented in Table 6, The Gambia 2013, Nigeria 2015, Rwanda 2013 and Senegal 2008 have higher mean users per net for conical nets than for rectangular, while the remaining countries have more users per rectangular net. The average cost for standard nets of various widths obtained from Global Fund in March 2016 (pers. comm.) was US$2.35 for rectangular and US$4.21 for conical (both CIF (Cost, Insurance and Freight). Rwanda’s population is roughly 12 million people. The country would need to procure about 6.6 million ITNs for a universal coverage campaign, using the ‘population divided by 1.8′ algorithm recommended by WHO [46, 47]. These parameters, inputted into the Excel tool, assume that the budget is fixed at 6.6 million ITNs x the cost of the standard net. The tool estimates that these 6.6 million standard nets would protect 10.2 million users, based on mean users per net, and that if alternative (conical) nets are procured, there would only be budget enough to procure 3.7 million of them instead of the needed 6.6 million. Despite higher mean users per conical net, total users if conical nets are purchased would be expected to be 6.1 million, an overall loss of 4.1 million users, due to the overall reduction in nets distributed. If the differential in mean users is calculated only in households that own both shapes (1.53), still just over 1.1 million users would be lost. In other words: conical nets would need to be less than 6% more expensive before any population-level gains in net users would be expected.

### Case study for size: Senegal

While size data are limited, the mean users per two-place net and three-place net from Senegal can be used to assess whether procuring three-place nets (the alternative net in this scenario) would be cost effective. Using dimensions of 180H × 160L × 150W as two-place or double, and 180H × 160L × 170W as three-place or queen size, with a cost differential of US$0.19, in Senegal’s case (population roughly 15 million, 7.5 million nets required), one would expect to see a net gain of about 750,000 net users if the three-place nets were procured, using the Excel tool. The increased mean users per larger net offsets an overall reduction of about 610,000 nets that could be procured.

## Discussion

While data are scarce on ITN attributes such as shape and size, the available data, taken together, indicate that user preferences for certain types of nets does not impede net use at the population level. It is worth noting that overall proportions of nets used the previous night are reasonably high (60–80%) and that the proportion of the population using nets if they have access to them is similarly high, with a mean use of ITNs given access of 81% across 81 surveys, and a median of 87%, as described in the ITN Access and Use Report [48].

It is telling that in only one survey were respondents asked about both their shape and colour preferences and the shapes and colours of the nets they own. It is not enough to simply ask respondents for their stated preferences, as this has no strong correlation with the rates at which the nets are used. What is observed is that people tend to use the nets they have, and that wealthier quintiles may seek out additional nets that meet their preferences. But preferences for shape and colour are not strong enough to impact net use rates overall, especially when taking into account the increased cost of conical ITNs.

In the multilevel model for Malawi 2014, having one’s preferred shape and colour net contributes to increased odds (1.78 OR) of using that net, but this association is not stronger than household supply of nets (having not enough, or more than enough), nor is it stronger than the net being an ITN. Average marginal effects were low (4.5%). Indeed, having only a net of one’s preferred shape (but not colour), or vice versa, was not significant in the model, demonstrating a relatively weak influence of preferences overall. Conical net shape improved the odds of the net being used by up to 2.26 times in Rwanda 2013, Rwanda 2014, Malawi 2010 and Malawi 2012. By 2014, however, shape alone ceased to be a significant predictor of net use in Malawi. Age of net was highly correlated (p < 0.001) with shapes and colours, but generally, net characteristics are aligned with distribution cohorts, and significant age results are likely a reflection of whenever the last campaign(s) occurred. Presence of holes and colour were not significant predictors of use.

The overall difference in the rates of use of nets of different shapes is minimal, ranging from 1 to 7 percentage points difference, and an average of 3.6 percentage points. The exception is Rwanda, where conical nets were used 14.3 percentage points higher than rectangular nets in 2013. While several of these small percentage point differences were statistically significant, due to the very large sample size of over 20,000 nets, these statistical differences in use rates should not be interpreted as programmatically significant.

In addition, when mean users under each type of net is calculated, and costs of procurement are taken into account, using the Excel tool, it is clear that even in Rwanda, given a fixed budget for nets, procuring the more expensive net results in a direct loss of overall ITN coverage which is not made up by the increased use of conical nets. Therefore, decisions to procure conical nets do not provide value for money under a fixed budget unless the price of conical nets is considerably reduced.

There is a significant influence of wealth quintile on both ownership and preferences for conical nets in particular. Wealthier households are more likely to own conical nets, more likely to own both conical and rectangular nets, and more likely to express preferences for conical nets. Procuring more expensive conical nets with public funds to satisfy the preferences of wealthier households goes against public health principles, especially when these are the very groups that have the means to purchase their preferred net if unsatisfied with the free rectangular option. Likewise, there is bias inherent in the observations in households owning both shapes where preferential use of conical nets over rectangular nets is seen (except in The Gambia), and the increased use of conical nets cannot therefore be extrapolated to all households.

The reasons for non-use of nets are well known and well described in Pulford et al. The primary reason for a net going unused the previous night are that it is not needed (extra), or because the perceived mosquito density is low, or because of heat [5]. These findings are corroborated with the data presented here. Among the many possible answers to the question ‘why did no one sleep under this net the previous night’, only a tiny proportion of respondents cite shape or size as the key problem. This again points to preferences being present, perhaps, but not key factors that determine net use. Likewise, making the effort to transform a net indicates a relatively strong level of shape preference. The fact that only 5% of nets in Senegal and 5% of conical nets in Malawi had been transformed indicates that the majority of the population in these settings are content to sleep under the net they have. Whether or not these types of alterations have any negative or positive impact on overall ITN durability remains to be studied.

Minimal data have been collected on preferences for textiles, although these seem relatively strong in Madagascar. There are no programmatically significant differences observed between textiles. There are relatively strong and uniform colour preferences in the available datasets, and as colour is not a key cost driver for ITNs [7], the current standard options of white, blue and green are adequate.

There are almost no data to assess the influence of size on net use. Anecdotally, taller nets are preferred as they provide more room to move around, more room for sexual activity and are easier to tuck in (Some of the preference for conical nets may in fact be due to their significantly taller height). Wider nets accommodate more sleepers. Indeed, given the increased mean users for wider nets, procuring these nets may be cost effective, given the relatively small increase in cost. However, procuring wider nets will not always result in increased users in each household, as a family of four, for example, may use one two-place net for the parents and one two-place net for the two children, and receiving three-place nets would not lead to additional individuals being protected. Likewise, several studies have noted that children age 6–15 years are the least likely to sleep under nets [49–51], both because they are less prioritized than small children and adults when nets are insufficient, and because they may not always be able to share sleeping spaces with siblings due to gender differences and associated cultural norms. Whether or not procuring wider nets can resolve these issues remains to be seen.

The variability of results over time in Malawi and in Rwanda demonstrate that preferences are not static. While stated preferences for conical nets in Malawi increased from 2010 to 2014, the strength of the association between conical shape and net use the previous night decreased over the same period. In Rwanda, conical nets were less likely to be used than rectangular nets in 2010, then significantly more likely than rectangular in 2013, and then less strongly in 2014. While it is possible that survey timing or sampling contributes to these changes, it is also likely that preferences do change over time, as households become accustomed to different types of nets. Both Guinea and Zimbabwe have recently procured rectangular nets after several rounds of conical nets (PMI, pers. comm.), and it will be important to assess use patterns after this transition.

The available data come mainly from sub-Saharan Africa, which has experienced in some countries, several rounds of mass ITN campaigns, and where retail markets and ability to purchase nets of one’s preference have been quite limited compared to Southeast Asia. These findings should not be extrapolated to Southeast Asia, where markets for nets are more vibrant and consumer preferences may have stronger influence over net use. Further research is needed to assess rates of net use for different net attributes in this region.

Additionally, the analysis of individual and household level data related to the effect of preferences for net type on the use of nets may be somewhat compromised by self-selection of households into ownership of the type of nets they are most likely to use. However, the limited market availability of varied types of nets may restrict this especially in rural areas of sub-Saharan Africa.

These analyses indicate that overall, at current price differentials, rectangular nets are a ‘best buy’ for the majority of the population and provide the most value for money. Given the trends in conical net preference (and purchase) by wealthier households, it is likely that the retail market is better suited to provide conical nets to these households than are donor organizations and Ministries of Health.

### Recommendations for measuring net preferences and their impact

There are several ways to approach measuring net preferences and their impact on net use. Each has its advantages and drawbacks. Methods 1–3 are not sufficient for informing national procurements; methods 4 is useful but not ideal, while method 5 is the recommended approach.

1. Small scale qualitative research on preferences: these studies, generally implemented using focus group discussions, with a limited sample in a restricted study area, have a tendency to focus largely on barriers to use when ITN use rates are relatively strong. These can make issues such as difficulty hanging nets or not having just the right shape and colour and size appear more important than they really are. Berthe et al. illustrate the concept nicely whereby focus group discussions often elicit vigorous complaints about nets despite high rates of actual use [33].
2. Small scale acceptability study: these studies tend to distribute ITNs of one or multiple types to a small number of households in a particular study area, usually at the initial stages of production or testing in order to confirm that the product is acceptable. However, the limited study area prevents extrapolation to the rest of the country, which becomes problematic for mass procurement, where the preferences of a small number of users would be applied to a nation as a whole. These types of studies may be better suited for particular vulnerable niche groups, or in Trials of Improved Practices (TIPS), for example among nomadic groups in sub-Saharan Africa, forest goers in Southeast Asia [52], and in South America [53]. Cluster-randomized, crossover acceptability studies, such as Atkinson [23] in the Solomon Islands, are a better approach than single acceptability studies, but have the same limitations in terms of national representativeness, and costs of ensuring national representation are likely to be prohibitive.
3. Household survey asking only about preferences: the results from this type of survey are able to assess net preferences across the country (if the survey is nationwide), but tell programmes absolutely nothing about whether or not these stated preferences have any influence on overall use of ITNs.
4. Household survey asking only about net shape, size: the results from these surveys provide valuable information on whether nets of various attributes are used differently, but cannot be linked to preferences which would show whether or not having a net of one’s preference is associated with an increased probability of being used. However, this type of data can be used to generate mean users per net of each type, which can then be used in the Excel Tool to calculate whether switching to the alternative net under a fixed budget would result in increased or decreased overall net use.
5. Household survey recording both preferences and matching net attributes: for countries that wish to justify procurement based on preferences, both these variables must be recorded and analysed. The implications of this are the addition of questions in the net roster on net shape and net size, and in the household questionnaire on key attributes (preferred shape, preferred size of net, preferred textile while it is easy to record shape and colour, width and length present challenges for measurement in the course of household interviews, as nets are not always available for measurement (e.g., not hanging, or tied up), and may not be easily categorized into single, double, queen, king, and other sizes (particularly for conical nets). Requesting that data collectors measure each net’s height length and width with a tape measure is likely too time consuming and consistently accurate measurements would be difficult. These studies could also be compromised by selection of households to net types that are preferred, which might bias estimates of the effect of preferences on net use.

### Limitations

These findings are limited by the lack of widespread data on net shape, width, colour, and length, all of which are key cost drivers, and by lack of nationally representative data on preferences for these attributes, most notably in Southeast Asia. In addition, despite massive scale-up of ITN distribution in the last decade, access to ITNs is still below target. Practically, this means that most people do not have a choice of nets to use within their household; they do not choose to sleep under a conical net or a square net on any given night; they choose to sleep or not sleep under the net (or nets) that they have access to. This limits household flexibility in using any preferred net, which this study has attempted to illustrate in the analyses on the households owning both shapes.

## Conclusions

Stated preferences for shape and colour do not significantly influence net use to degrees that would require action by programme planners. By and large, people are using the nets they receive, and when they do not, it is for reasons unrelated to shape and size (primarily perceived mosquito density, heat or an excess of nets). Wealthier quintiles tend to own greater numbers of conical nets, indicating that they have the ability to obtain or purchase these nets on their own, and wealthier quintiles also use conical nets preferentially. However, the preferences and use patterns of a sub-set of wealthier households should not drive procurement policies, as the increased manufacturing costs for conical nets are not outweighed by the very small, often non-existent, increases in use rates. Programmes that wish to explore the relationship between net attributes, preferences and use rates should include these questions in nationally representative household surveys to be able to capture trends across geographic and socio-economic groups.

## Additional files

**Additional file 1.** Literature reviewed.

**Additional file 2.** Determinants of shape preferences.

**Additional file 3.** Ownership of both conical and rectangular nets across region and wealth quintile.

**Additional file 4.** Excel tool for cost and utility function calculation. This file provides instructions for calculating mean users per net from DHS and MIS datasets, and an excel-based tool to calculate increases or decreases in ITN users, depending on the cost differentials of different nets and the differentials in mean users per nets of different attributes.
