# Supplementary material for: Effect of user preferences on ITN use: a review of literature and data
Source: Malar J. 2017 Jun 1;16:233. doi: 10.1186/s12936-017-1879-8 (PMC5455118; doi:10.1186/s12936-017-1879-8)
Supplement: Supplementary file 1 — Additional file 1. Literature reviewed. [file 12936_2017_1879_MOESM1_ESM.docx]

**Additional File 1 Literature reviewed**

|  | Author | Article title | Year | Country | Type of study | Methodology | Type of preference (stated or net use rate) |
| --- | --- | --- | --- | --- | --- | --- | --- |
|  | MacCormack | Gambian cultural preferences in the use of insecticide-impregnated bed nets | 1986 | Gambia | Simple preferences | Direct observation and interviews | Stated |
|  | McCormack | The human host as active agent in malaria epidemiology | 1987 | Gambia | Conference paper | Direct observation and interviews | Stated |
|  | MacCormack | Use of insecticide-impregnated bed nets in Gambian primary health care: economic aspects | 1989 | Gambia | Acceptability | Direct observation and interviews | Stated |
|  | Aikens | A malaria control trial using insecticide-treated bed nets and targeted chemoprophylaxis in a rural area of The Gambia, west Africa. 4. Perceptions of the causes of malaria and of its treatment and prevention in the study area. | 1993 | Gambia | Simple preferences | Cross sectional survey; focus group discussions | Stated |
|  | Gyapong | Introducing insecticide impregnated bednets in an area of low bednet usage: an exploratory study in north‐east Ghana | 1996 | Ghana | Simple preferences | Direct observation and interviews | Stated |
|  | Binka | Acceptability and use of insecticide impregnated bednets in northern Ghana. | 1997 | Ghana | Simple preferences | Cross-sectional survey; focus group discussions | Stated |
|  | Bean | OXFAM GB Malaria Control Assessment Upper Nile - South Sudan 16 Apr -31 May 2001 | 2001 | Sudan | Simple preferences | Field report (unpublished) | Stated |
|  | Tami | Evaluation of Olyset insecticide-treated nets distributed seven years previously in Tanzania | 2004 | Tanzania | Simple preferences | Cohort followup | Stated |
|  | Das | Population preference of net texture prior to bed net trial in Kala-Azar-endemic areas | 2007 | India/  Nepal | Crossover acceptability | Cross sectional survey; focus group discussions | Stated |
|  | Shirayama | Maintenance behaviour and long-lasting insecticide-treated nets (LLITNs) previously introduced into Bourapar district, Khammouane province, Lao PDR. | 2007 | Laos | Simple preferences | Cross-sectional survey | Stated |
|  | Fernando | Community factors affecting long-lasting impregnated mosquito net use for malaria control in Sri Lanka. | 2008 | Sri Lanka | Simple preferences | Cross-sectional survey | Stated |
|  | Harvey | The whole world will be able to see us: determining the characteristics of a culturally appropriate bed net among mestizo communities of the Peruvian Amazon. | 2008 | Peru | Simple preferences | In depth interviews and ranking exercise | Stated |
|  | Atkinson | A qualitative study on the acceptability and preference of three types of long-lasting insecticide-treated bed nets in Solomon Islands: implications for malaria elimination | 2009 | Solomon Islands | Simple preferences | Focus group discussions | Stated |
|  | Atkinson | A cluster randomized controlled cross-over bed net acceptability and preference trial in Solomon Islands: community participation in shaping policy for malaria elimination | 2009 | Solomon Islands | Crossover acceptability | 3 stage cluster, 10 days per net brand crossover | Stated |
|  | Baume | Factors associated with use and non-use of mosquito nets owned in Oromia and Amhara Regional States, Ethiopia | 2009 | Ethiopia | Determinants of use | Cross-sectional survey in 23 communities | Use rate |
|  | Ng'ang'a | Bed net use and associated factors in a rice farming community in Central Kenya. | 2009 | Kenya | Simple preferences | Cross-sectional survey in 4 villages | Stated |
|  | Atkinson | Community participation for malaria elimination in Tafea Province, Vanuatu: Part I. Maintaining motivation for prevention practices in the context of disappearing disease | 2010 | Vanuatu | Simple preferences | Focus group discussions and key informant interviews | Stated |
|  | Banek | Evaluation of Interceptor long-lasting insecticidal nets in eight communities in Liberi | 2010 | Liberia | Acceptability | Cross-sectional survey in 8 rural villages | Stated |
|  | Beer | System effectiveness of a targeted free mass distribution of long lasting insecticidal nets in Zanzibar, Tanzania | 2010 | Tanzania | Determinants of use | Cross sectional survey in 2 districts | Use rate |
|  | Ndjinga | The importance of education to increase the use of bed nets in villages outside of Kinshasa, Democratic Republic of the Congo | 2010 | DRC | Determinants of use | Cross-sectional survey in 2 villages | Use rate |
|  | Baume | Predictors of mosquito net use in Ghana. | 2011 | Ghana | Determinants of use | Cross-sectional survey | Use rate |
|  | Lover | An exploratory study of treated-bed nets in Timor-Leste: patterns of intended and alternative usage | 2011 | Timor-Leste | Simple preferences | Focus group discussions | Stated |
|  | Gobena | Low long-lasting insecticide nets (LLINs) use among household members for protection against mosquito bite in Eastern Ethiopia | 2012 | Ethiopia | Other | Cross-sectional survey | Stated |
|  | Macintyre | Determinants of hanging and use of ITNs in the context of near universal coverage in Zambia. | 2012 | Zambia | Determinants of use | MIS-style cross-sectional survey | Use rate |
|  | Sande | An investigation of the use of rectangular insecticide-treated nets for malaria control in Chipinge District, Zimbabwe: a descriptive study | 2012 | Zimbabwe | Simple preferences | Cross-sectional survey | Stated |
|  | Peeters Grietens | Traditional nets interfere with the uptake of long-lasting insecticidal nets in the Peruvian Amazon: the relevance of net preference for achieving high coverage and use | 2013 | Peru | Determinants of use | Direct observation and interviews; survey; case-control | Use rate |
|  | Aleme | Willingness to pay for insecticide-treated nets in Berehet District, Amhara Region, Northern Ethiopia: implication of social marketing | 2014 | Ethiopia | Simple preferences | Cross-sectional survey | Stated |
|  | Berthe | When I sleep under the net, nothing bothers me; I sleep well and I'm happy: Senegal's culture of net use and how inconveniences to net use do not translate to net abandonment | 2014 | Senegal | Simple preferences | Focus group discussions | Stated |
|  | Birhanu | Access to and use gaps of insecticide- treated nets among communities in Jimma Zone, southwestern Ethiopia: baseline results from malaria education interventions | 2015 | Ethiopia | Simple preferences | Cross sectional survey; focus group discussions; key informant interviews | Stated |
|  | Gore-Langton | Investigating the acceptability of non-mesh, long-lasting insecticidal nets amongst nomadic communities in Garissa County, Kenya using a prospective, longitudinal study design and cross-sectional household surveys | 2015 | Kenya | Acceptability | Prospective longitudinal acceptability trial with focus group discussions and key informant interviews | Stated |
|  | Gryseels | Re-imagining malaria: heterogeneity of human and mosquito behaviour in relation to residual malaria transmission in Cambodia | 2015 | Cambodia | Simple preferences | Direct observation and interviews | Stated |
|  | Whidden | Patterns and predictive factors of long-lasting insecticidal net usage in a previously high malaria endemic area in Sri Lanka: a cross-sectional survey | 2015 | Sri Lanka | Determinants of use | Cross-sectional study | Use rate |
|  | Mattern | “Tazomoka Is Not a Problem”. Local Perspectives on Malaria, Fever Case Management and Bed Net Use in Madagascar | 2016 | Madagascar | Simple preferences | Direct observation and interviews | Stated |
