# Supplementary material for: Effect of user preferences on ITN use: a review of literature and data
Source: Malar J. 2017 Jun 1;16:233. doi: 10.1186/s12936-017-1879-8 (PMC5455118; doi:10.1186/s12936-017-1879-8)
Supplement: Supplementary file 2 — Additional file 2. Determinants of shape preferences. [file 12936_2017_1879_MOESM2_ESM.docx]

**Additional File 2 Determinants of shape preferences**

As a frequent complaint of hanging rectangular nets is that it requires four, rather than one hole in the wall or ceiling, one hypothesis is that shape preference is at least partially driven by the hardness of one’s walls. Therefore, a multivariate logistic regression was used to assess determinants of a household’s reported shape preference in five surveys. When controlling for urban residence, wealth quintile, the gender of the respondent, the age of the household head, and region, the type of wall is only a significant determinant of conical shape preference in Malawi 2014. Wealth quintile is the strongest predictor of conical shape preference. Female respondents were more likely to prefer conical shape than males except in Malawi 2014. In Kenya, Coast and Northeastern region had higher odds of conical shape preference than other regions.

**Table S1 Adjusted odds ratios predicting preference for conical shaped nets in five surveys across four countries in sub-Saharan Africa**

|  | Guinea 2012 | Malawi 2010 | Malawi 2014 | Madagascar 2013 | Kenya 2015 |
| --- | --- | --- | --- | --- | --- |
| Urban residence | 0.90 | 0.67*** | 0.54** | 1.089 | 0.89 |
|  |  |  |  |  |  |
| Wealth quintile (ref: Poorest) |  |  |  |  |  |
| Poorer | 1.34* | 1.18** | 1.12 | 1.41 | 1.40** |
| Middle | 1.80*** | 1.28*** | 1.44 | 1.53* | 1.57** |
| Richer | 2.00*** | 1.65*** | 1.41 | 1.79** | 1.61** |
| Richest | 2.62*** | 3.05*** | 3.84*** | 2.19*** | 1.26 |
|  |  |  |  |  |  |
| Age of household head (ref: 16-35) |  |  |  |  |  |
| 35-49 | 1.08 | 1.02 | 1.49* | 0.74*** | 0.94 |
| 50+ | 0.96 | 0.83*** | 1.00 | 0.54*** | 0.80* |
|  |  |  |  |  |  |
| Respondent is female (*vs* male) | 1.21** | 1.11** | 1.00 | 1.37*** | 1.50*** |
|  |  |  |  |  |  |
| Type of walls (ref: Wood/mud) |  |  |  |  |  |
| Stone/cement | 1.07 | 1.10 | 1.58* | 1.02 | 1.10 |
|  |  |  |  |  |  |
| Region (Ref: A) |  |  |  |  |  |
| B | 1.18 | 0.88 | 0.82 | 0.86 | 1.84* |
| C | 0.89 | 1.09 | 1.15 | 0.60* | 0.57** |
| D | 1.52* |  |  | 0.62** | 0.64** |
| E | 1.54 |  |  |  | 0.59** |
| F |  |  |  |  | 0.68* |
| G |  |  |  |  | 0.62* |
| H |  |  |  |  | 0.44*** |

Regions:

Guinea: A-Conakry, B-Basse Guinée, C-Moyenne Guinée, D-Haute Guinée, E-Guinée Forestiere;
Malawi: A-Northern, B-Central, C-Southern;
Madagascar: A-Equatorial, B-Tropical, C-Sub-desert, D-High Plateau;
Kenya: A-Coast, B-Northeastern, C-Eastern, D-Central, E-Rift Valley, F-Western, G-Nyanza, H-Nairobi.
