# Supplementary material for: Effect of user preferences on ITN use: a review of literature and data
Source: Malar J. 2017 Jun 1;16:233. doi: 10.1186/s12936-017-1879-8 (PMC5455118; doi:10.1186/s12936-017-1879-8)

**Additional File S3 Ownership of both conical and rectangular nets across region and wealth quintile**

**Table S2 Proportion of households that own both conical and rectangular nets by region, in eight countries**

| **Survey** | **Regions** | | | | | | | | | | | | | | |
| --- | --- | --- | --- | --- | --- | --- | --- | --- | --- | --- | --- | --- | --- | --- | --- |
| **Burkina Faso** | Boucle de mouhoun | Cascades | Centre | Centre-Est | Centre-Nord | Centre-Ouest | Centre-Sud | Est | Hauts Basins | Nord | Plateau | Sahel | Sud-Ouest |  | Total |
| **2014** | 0.96 | - | 2.06 | 0.55 | - | 0.13 | 1.33 | 0.60 | 1.29 | 1.03 | - | - | 0.59 |  | 0.82 |
| **Gambia** | Banjul | Kanifing | Brikama | Mansakon | Kerewan | Kuntaur | Janjanbu | Basse |  |  |  |  |  |  | Total |
| **2013** | 25.65 | 24.16 | 29.61 | 46.35 | 38.85 | 45.33 | 50.45 | 53.61 |  |  |  |  |  |  | 37.88 |
| **Malawi** | Northern | Central | Southern |  |  |  |  |  |  |  |  |  |  |  | Total |
| **2010** | 22.46 | 18.12 | 18.92 |  |  |  |  |  |  |  |  |  |  |  | 19.06 |
| **2012** | 16.4 | 10.49 | 12.89 |  |  |  |  |  |  |  |  |  |  |  | 12.36 |
| **2014** | 9.44 | 17.3 | 15.34 |  |  |  |  |  |  |  |  |  |  |  | 14.84 |
| **Mali** | Kayes | Koulikoro | Sikasso | Segou | Mopti | Bamako |  |  |  |  |  |  |  |  | Total |
| **2015** | 6.54 | 8.58 | 6.67 | 7.47 | 2.80 | 14.35 |  |  |  |  |  |  |  |  | 7.70 |
| **Rwanda** | Kigali | South | West | North | East |  |  |  |  |  |  |  |  |  | Total |
| **2010** | 33.24 | 29.2 | 24.14 | 19.15 | 31.33 |  |  |  |  |  |  |  |  |  | 27.95 |
| **2013** | 32.53 | 36.86 | 31.75 | 31.59 | 34.36 |  |  |  |  |  |  |  |  |  | 33.77 |
| **2014** | 10.56 | 17.87 | 21.35 | 22.55 | 18.52 |  |  |  |  |  |  |  |  |  | 18.42 |
| **Senegal** | Dakar | Ziguinchor | Diourbel | Saint-Louis | Tambacounda | Kaolack | Thiés | Louga | Fatick | Kolda | Matam | Kaffrine | Kedougou | Sedhiou | Total |
| **2008** | 21.42 | 26.14 | 9.156 | 18.03 | 20.24 | 23.89 | 25.6 | 32.59 | 30.04 | 23.66 | 20.95 |  |  |  | 23.15 |
| **2010** | 33.88 | 21.34 | 48.23 | 35.46 | 26.23 | 60.61 | 21.64 | 36.83 | 21.41 | 20.15 | 25.95 | 24.64 | 11.37 | 11.88 | 32.43 |

**Figure S1: Proportion of net-owning households that own both conical and rectangular nets, by wealth quintile, in eight countries**


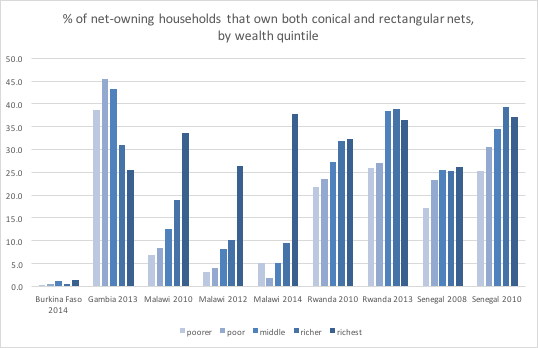

Supplement: Supplementary file 3 — Additional file 3. Ownership of both conical and rectangular nets across region and wealth quintile. [file 12936_2017_1879_MOESM3_ESM.docx]
